# 

**Published:** 1908-01-15

**Authors:** 


					﻿Cental
Register
NELVILLE S. HOFF, Editor,
Ann Arbor, Mich.
Vol. LXII — JANUARY, 1908— No. 1
SAMUEL A. CROCKER & CO., Publishers
OHIO DENTAL AND SURGICAL DEPOT
35 W. FIFTH STREET, CINCINNATI, 0.
/
PRICE, ONE DOLLAR A YEAR
Entered at the Posloffice at Cincinnati, O., as second-class mail mattei
				

